# Intestinal allergic inflammation in birch pollen allergic patients in relation to pollen season, IgE sensitization profile and gastrointestinal symptoms

**DOI:** 10.1186/2045-7022-4-19

**Published:** 2014-05-30

**Authors:** Georgios Rentzos, Vanja Lundberg, Per-Ove Stotzer, Teet Pullerits, Esbjörn Telemo

**Affiliations:** 1 Section of Allergology, Sahlgrenska University Hospital, Gothenburg 413 45, Sweden; 2 Department of Rheumatology and Inflammation Research, Sahlgrenska Academy, University of Gothenburg, Gothenburg, Sweden; 3 Department of Internal Medicine, Section of Gastroenterology, Sahlgrenska University Hospital, Gothenburg, Sweden

**Keywords:** Birch-pollen, Gastrointestinal symptoms, Allergic inflammation, Gastroscopy, Biopsies, Allergen components

## Abstract

**Background:**

Birch pollen allergic patients frequently experience gastrointestinal upset accompanied by a local allergic inflammation in the small intestine especially during the pollen season. However, it is not known if the GI pathology is connected to the subjective symptoms of the patient. The objective of this study was to evaluate the immune pathology of the duodenal mucosa and the serum IgE antibody profiles in birch pollen allergic patients in relation to their gastrointestinal symptoms, during and outside the birch pollen season.

**Methods:**

Thirty-two patients with birch pollen allergy and sixteen healthy controls were enrolled in the study. Twenty allergic patients had gastrointestinal symptoms and twelve did not. All participants underwent an allergy investigation and gastroscopy with duodenal biopsy. The duodenal biopsies were retrieved during the pollen season (May-June) and off-season (November-March). The biopsies were immunostained for mast cells (IgE and tryptase), eosinophils, T cells (CD3), and dendritic cells (CD11c). Pollen-specific IgE antibodies were determined by ImmunoCAP and component microarray (ISAC).

**Results:**

Patients in both pollen allergic groups showed similar degree of intestinal allergic inflammation during the pollen season regardless of gastrointestinal symptoms. The eosinophils, mast cells and dendritic cells were increased in the mucosa. Patients with gastrointestinal symptoms had significantly elevated IgE antibodies to birch (rBet v 1), hazelnut (rCor a 1), and apple (rMal d1) during the pollen season.

**Conclusions:**

Patients allergic to birch pollen have clear signs of an ongoing allergic inflammation in their intestinal mucosa, which is aggravated during the pollen season. The magnitude of the allergic intestinal inflammation is not associated with subjective gastrointestinal symptoms of the individual patient.

## Background

Patients allergic to pollen may in addition to their respiratory symptoms also experience gastrointestinal disturbances, particularly during the pollen season. We have previously reported that there are clear histological signs of an ongoing allergic gastrointestinal inflammation in birch pollen allergic patients who have gastrointestinal symptoms during the birch pollen season [1]. The pathophysiological mechanisms underlying this type of allergic reaction is still obscure [2-4], and it is unclear whether the gastrointestinal symptoms are connected to the intestinal inflammation.

The nature of the gastrointestinal (GI) symptoms in pollen allergic patients varies. Some patients experience gastrointestinal symptoms only during the pollen season while others have continuous symptoms that aggravate during the pollen season. Probably some of the latter patients are diagnosed with irritable bowel syndrome (IBS). Indeed, it has been shown before that there is an ongoing inflammation in the duodenal mucosa in atopic IBS patients that is not seen in non-atopic IBS patients [5-7]. Interestingly, many patients with eosinophilic esophagitis were diagnosed during the pollen season [8-10]. Whether the pollen exposure by itself or rather the ingestion of birch-pollen related food has an impact on the gastrointestinal inflammation is not known.

An association between increased intestinal permeability and severe bronchial hyperreactivity or asthma has been indicated [11,12]. Patients with asthma or atopy without any GI symptoms showed an increase of inflammatory mediators (IL-3, IL-5, GM-CSF), in the expanded populations of eosinophils and mast cells seen in the duodenal biopsies [13]. Furthermore, in children, a relation between allergy to foods and severity of asthma has been demonstrated [14], and asthmatic patients may experience life threatening exacerbation when allergy to different foods co- exists [15].

A possible connection between the gastrointestinal symptoms and IgE sensitization profile can be monitored by a relatively new diagnostic tool, the allergen microarray chip ISAC (Thermo Fisher/Phadia). This test allows profiling of IgE reactivity to well defined allergen components from different sources, which enables a more precise monitoring of the IgE sensitization pattern to food allergen components that are related to birch- pollen [16,17].

The aim of this study was to explore the immune pathology of the duodenal mucosa in birch pollen allergic patients, with or without GI symptoms, outside and during the birch pollen season. In addition, we aimed to relate these findings to the IgE antibody profile against birch related food allergen components in these patients. The results may add to the understanding of the etiology of GI symptoms in pollen allergic patients.

## Materials and methods

### Study population

Patients and healthy controls were recruited from the Asthma and Allergy clinic at the Sahlgrenska University Hospital in Gothenburg, Sweden. The patients were requested to answer a detailed questionnaire about their birch pollen related symptoms and if and when they had gastrointestinal symptoms (supplement attached).

**Figure 1:**
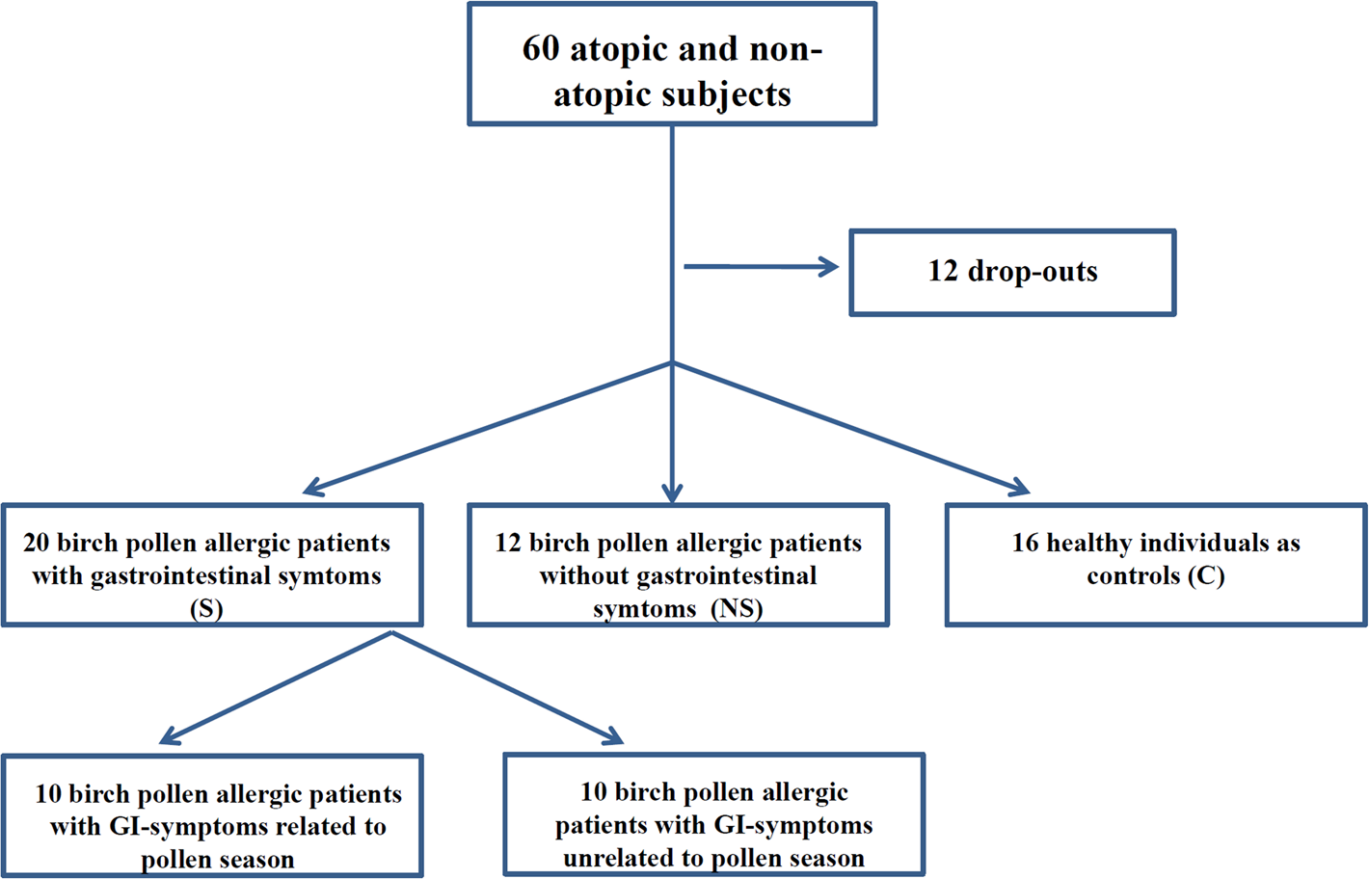
Overview of the selected study populations.

All patients included were between 18–50 years old and allergic to birch pollen. Some of the patients were also allergic to other allergens (Table 1), and some were diagnosed with oral allergy syndrome (OAS) and/or asthma (Table 2). The diagnosis of birch-pollen allergy was confirmed by a history of rhinoconjuctivitis and a positive skin prick test for birch pollen. In total 60 subjects were recruited to the study, twenty in each group. There were 12 drop-outs, mainly due to discomfort during the gastroscopy, and 48 subjects remained in the study. Patients in the first group (S) (n = 20) were allergic to birch pollen and had gastrointestinal symptoms during the pollen. Patients in the second group (NS) (n = 12) were also allergic to birch pollen but had no gastrointestinal symptoms during the pollen season. The third group (C) (n = 16) included healthy non-allergic individuals with negative skin-prck test (SPT) and IgE-tests for inhalation allergens, and served as controls (Figure 1). Exclusion criteria for all subjects were confirmed inflammatory bowel disease, celiac disease (none of the subjects were positive for transglutaminase or gliadin antibodies in serum) or other gastroenterological disease, food allergy to staple foods (egg-white, milk, wheat, soy, peanut, codfish), pregnancy, lactation, rheumatic or systemic disease, immune deficiency, previous or current treatment with immunotherapy. The gastroscopies were performed only as part of the present study. All gastroscopies were executed by experienced gastroenterologists, and no signs of macroscopic pathology or abnormality were found neither in the esophageal nor in the gastric or duodenal mucosa in any of the patients.

**Table 1:** Demographic data, and sensitization frequency to inhalant allergens

	**S (n = 20)**	**NS (n = 12)**	**C (n = 16)**
**Men**	10	7	5
**Women**	10	5	11
**Age (mean)**	37	33	33
**Sensitization frequency**			
**Birch**	20	12	
**Grass**	15	7	
**Mugwort**	6	6	
**Animal dander**	11	3	
**Dust mites**	5	2	
**IgE-reactivity (median)**			
**Total IgE**	60.00 kU/L	58.00 kU/L	15.00 kU/L
**IgE birch**	6.14 kU/L	3.90 kU/L	0.00 kU/L
**IgE grass**	3.20 kU/L	3.65 kU/L	0.00 kU/L
**IgE mugwort**	0.05 kU/L	0.20 kU/L	0.00 kU/L

Median IgE values for pollen allergens and total IgE in the subjects included in the study are depicted. NS = no gastrointenstinal symptoms, S = with gastrointenstinal symptoms, C = healthy controls).

**Table 2:** The number of patients with asthma and OAS (oral allergy syndrome) and the frequency of the most frequent gastrointestinal symptoms

	**S (n = 20)**	**NS (n = 12)**	
**Asthma**	11	3	
**OAS**	12	6	
**Gastrointestinal symptoms**	**S (n = 20)**		
Pollen season-related symptoms			10
Symptoms all the time			10
**Most frequent symptoms**	**(%)**		
Abdominal distension			78
Gases			72
Stomach pain			44
Diarrhea			44
Constipation			33
Pain in the lower bowel			28
Bowel cramps			28
Pain relief with defecation			22
Burning sensation in esophagus			22
Nausea			11
Pain related to meal			11

Median IgE values for pollen allergens and total IgE in the subjects included in the study are depicted. NS = no gastrointenstinal symptoms, S = with gastrointenstinal symptoms, C = healthy controls).

The demographic and clinical characteristics of the patients and controls are shown in Table 1.

All allergic patients were under intermittent medication with anti-histamines, nasal steroids and eye-drops during the pollen-season. Patients with asthma used inhaled corticosteroids and beta-mimetics when required. None of the patients needed medication with oral or injected corticosteroids.

The study was approved by the Ethics Committee of Regional Ethical Review Board in Gothenburg with Dnr. 452–06. Written Informed consent was obtained from the subjects.

### Allergy assessment

#### ImmunoCAP

The serum levels of total IgE and IgE antibodies to a mix of common food allergens (fx5; egg-white, milk, wheat, soy, peanut, codfish) and pollen allergens (birch, grass, mugwort) were measured using ImmunoCAP (Thermo Fisher/Phadia, Uppsala, Sweden) according to the manufacturer’s instructions.

#### ISAC

Blood samples were collected during the pollen season (5^th^ May until 10^th^ June) and outside the birch pollen season (1^st^ October until 5^th^ March) for the determination of IgE against allergen components using the allergen microarray immunoassay, ImmunoCAP ISAC (Thermo Fisher/Phadia) on which 103 allergen components are spotted. Analysis included reactivity to the major Birch allergens and the rest of PR-10 group of proteins, and the major allergen from Timothy rPhl p 1. The results are expressed as ISAC Standardized Units (ISU) with a threshold of ≥0.3 ISU. The ISAC analysis was performed as recommended by the manufacturer. The analysis of the data was focused on the PR-10 proteins and birch- related foods and a mean sensitization score was used.

All analyses except ISAC were performed at the Laboratory of Clinical Biochemistry, Sahlgrenska University Hospital, Gothenburg. The ISAC analyses were carried out at the Department of Clinical Immunology, Karolinska University Hospital, Stockholm, Sweden.

### Gastroscopy and duodenal biopsies

Gastroscopies and duodenal biopsy sampling were performed by gastroenterologist at the Department of Endoscopy in the gastroenterology clinic, Sahlgrenska University Hospital during the pollen season between the 5^th^ of May until 5^th^ of June, and outside the pollen season between 1^st^ November and 5^th^ of March. The patients had to avoid intake of food and liquids for at least 12 hours before the gastroscopy. Gastroscopies were performed with Olympus GIF Q160, Q180 or Fujinon 450 WR5 endoscopes. Biopsy specimens (6–7 specimens 2 to 3 mm in size) from the descending part of the duodenum were obtained. The tissue specimens were immediately embedded in O.C.T. (Optimal Cutting Temperature) compound (Tissue-Tek Miles, Inc, Elkhart, In, USA), frozen in isopentane precooled by liquid nitrogen, and finally transferred into liquid nitrogen. The biopsy specimens and sera were kept at -70°C until analyzed. All gastroscopies were performed during three consecutive years 2008–2010.

### Immunohistochemistry

Cryostat sections (7 μm) were stained as previously described [18,19]. The slides were coded and evaluated in a random order. All sections, except the ones detecting eosinophilic endogenous peroxidase, were blocked by the glucose oxidase sodium azide method to quench endogenous peroxidase activity [20]. Eosinophilic endogenous peroxidase was detected by just adding AEC-substrate.Mouse monoclonal antibodies with the following specificities were used: IgE (CIA-E-7.12, IgG1 κ, DAKO, Stockholm, Sweden), CD3 (UCHT1, IgG1 κ, SIGMA, St Louis, Mo, USA), mast cells tryptase (EC 3.4.21.59 Clone AA1, DacoCytomation, Glostrup, Denmark ) and CD11c purified (Integrin alpha X, p150/95, leu M5 alpha Clone 3.9 eBioscience, San Diego, Ca, USA). As a secondary antibody biotinylated horse anti-mouse IgG (H + L) (BA-2000, Vector Laboratories, Burlingame, Ca, USA) was used followed by an avidin-biotin-peroxidase enzyme complex (Vectastain ABC elite, Vector, UK). The slides were developed by adding AEC substrate and counter stained with Mayer’s Hematoxylin. Positively stained cells were counted by using a computer supported image analysis system Leica Q500MC (Leica, Cambridge, United Kingdom). Cells in at least five 200 × fields from the villi and the basal lamina propria regions of the mucosa were counted by a blinded observer. The IgE, CD3 and tryptase positive cells were recorded as the number of stained cells/mm^2^ of tissue. The density of CD11c + dendritic cells was estimated as the relative stained area in at least five 100 × fields and expressed as percent stained tissue area.

### Statistics

The values represent individual data points or means and median values. The statistical analyses were carried out by using SPSS Statistics 17.0. Data are reported as medians with interquartile ranges (IQR), and Mann–Whitney U-tests were used for statistical comparison between groups of patients. Data for each individual on cell counts in biopsies and IgE reactivity between the seasons was compared by Wilcoxon signed rank test. Correlations between different parameters within the same group were evaluated by Spearman’s correlation coefficient or, after log-transformation, with Pearson’s correlation coefficient. All tests were two-tailed and the level of significance was set to P < 0.05.

## Results

### Questionnaire

Half of the S-patients (n = 10) reported symptoms only during the pollen season while the rest had subjective symptoms without any seasonal variation. Only one patient experienced worsening of symptoms when ingesting birch pollen-related foods. The most frequent symptoms were: abdominal distension (73%) gases (72%), stomach pain (44%), diarrhea (44%), constipation (33%). Clinical and demographic characteristics of the subjects are shown in Table 2. There were no significant differences in the frequency and severity of symptoms that could be related to the severity of the pollen seasons. The data concerning the pollen counts between 2007–2010 are provided in the Additional file 1: Figure S1.

### Immunohistochemical parameters of duodenal mucosal

#### Allergic inflammation in the duodenal mucosa in birch pollen allergic patients compared to healthy controls

**Figure 2:**
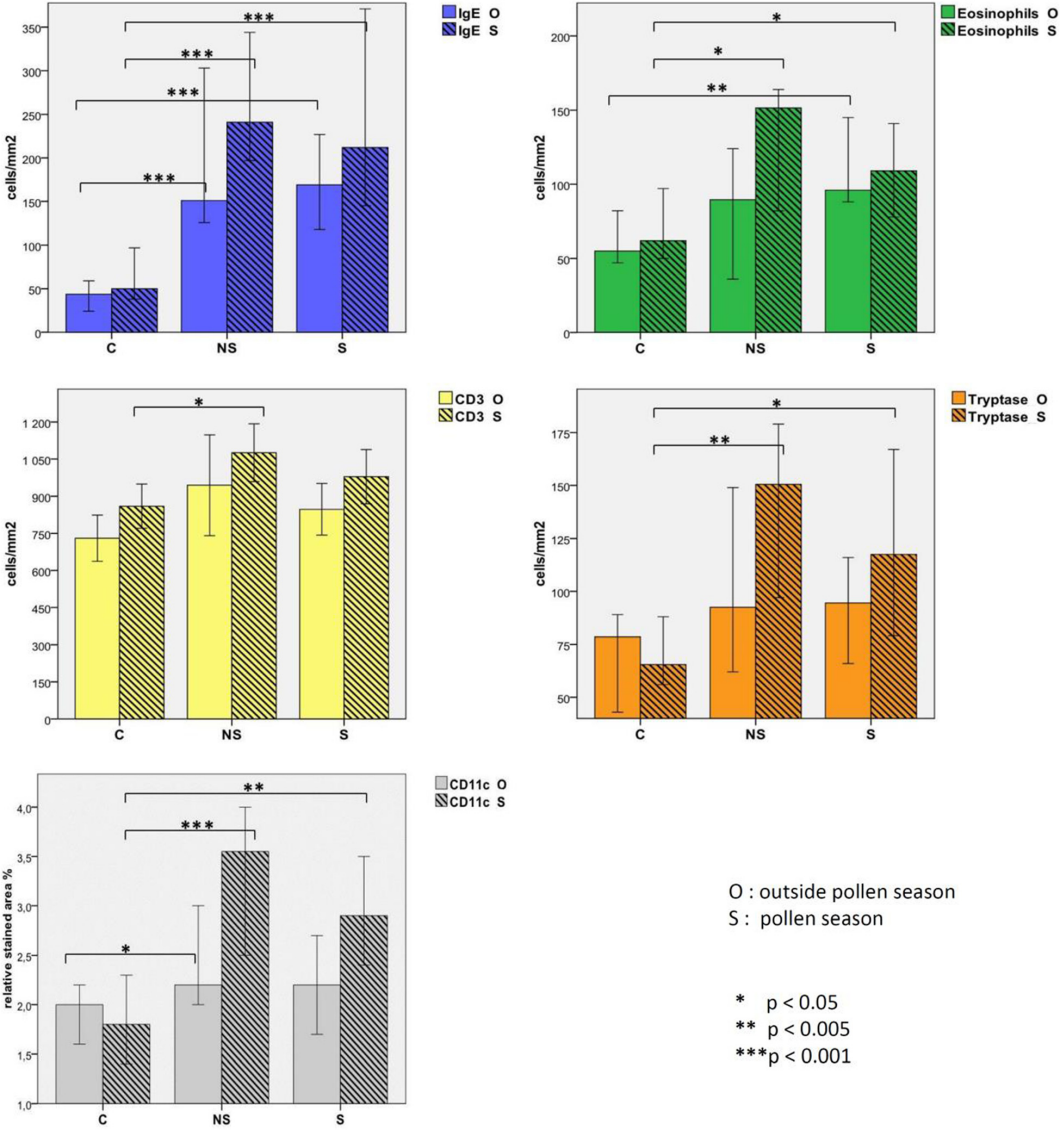
**The frequency (median and 95% confidence interval) of immune cells in the duodenal mucosa in the different patient groups as indicated on the x axis (C = healthy controls, NS = patients without GI symptoms, S = patients with GI symptoms).** The filled bars represent biopsies taken outside the birch pollen season and the striped bars during the birch pollen season.

The numbers of IgE-positive cells, eosinophils, tryptase-positive cells and CD11c + dendritic cells were significantly higher in S-patients compared with controls in the biopsies taken during the pollen season. However, outside the pollen season only IgE-positive cells and eosinophils were significantly higher in the S-patients. In the NS-patients the biopsies taken during the pollen season showed significantly elevated number of IgE-positive cells, eosinophils, CD3+ T cells, tryptase- positive cells and CD11c + dendritic cells as compared with the controls. In this group only IgE-positive cells and CD11c + dendritic cells remained elevated outside the pollen season (Figure 2).

#### Intestinal allergic inflammation in birch pollen allergic patients with or without GI symptoms

**Figure 3:**
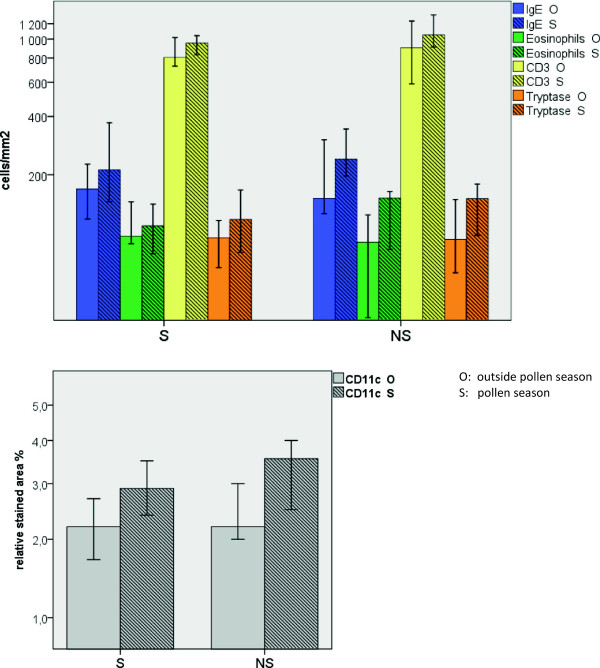
**Seasonal variation in the duodenal cell count (median cell count per mm**^**2 **^**and 95% confidence interval) in patients with (S) or without (NS) gastrointestinal symptoms.** The filled bars represent biopsies taken outside the birch pollen season and the striped bars during the birch pollen season.

There was no significant difference in the duodenal cell counts (mast cells, eosinophils, T cells, and CD11c + dendritic cells) between the patient group with gastrointestinal symptoms (S) as compared to patient group without GI symptoms (NS). This was true both for samples taken during and outside the pollen season (Figure 3).

### Seasonal variation in duodenal allergic inflammation

#### Patients with gastrointestinal symptoms (S-group)

**Figure 4:**
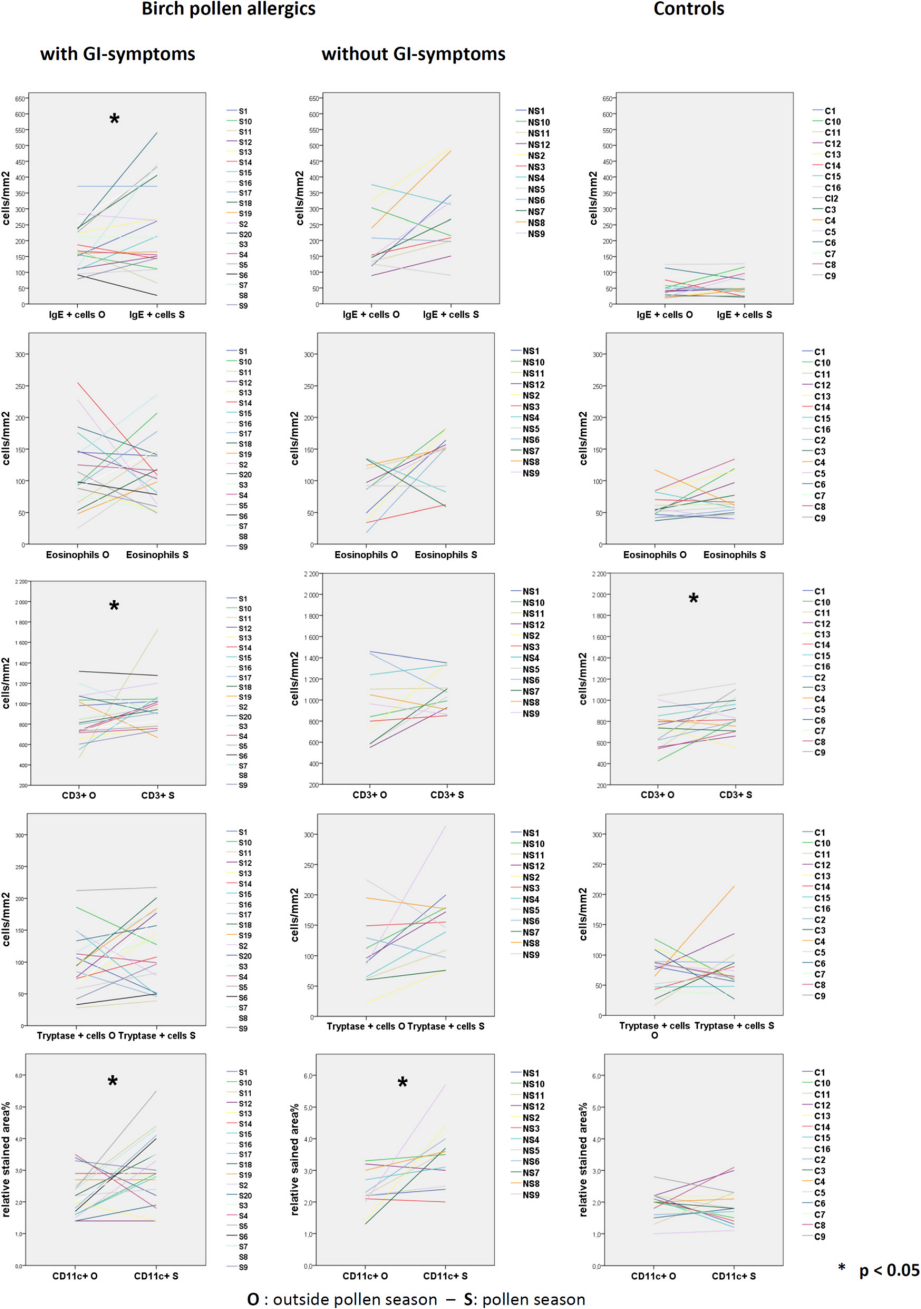
**Seasonal variation in the duodenal cell count in individual patients of the different study populations.** (C = healthy controls, NS = patients without GI symptoms, S = patients with GI symptoms). Each colored line represents one patient and the left value is from outside the birch pollen season and the value to the right from samples taken during the birch pollen season.

During the birch pollen season there were significantly higher numbers of IgE-positive cells (median: 212 cells/mm^2^) (p = 0.049), CD3+ T cells (median: 954.50 cells/mm^2^) (p = 0.040) and CD11c + dendritic cells (median: 2.9% of relative stained area) (p = 0.023) in patients with GI symptoms than in the same patients outside the pollen season. (Figure 4). However, there was no difference between patients who experienced pollen season related gastrointestinal symptoms (n = 10) compared to patients with symptoms not related to season (n = 10) in the S-group. There was a correlation between the total serum IgE and IgE-positive cells in the biopsies taken during the pollen season (r = 0.540, p = 0.014). In contrast, there was no correlation between the duodenal cell counts and the total number of symptoms regardless of season. Furthermore, patients with oral allergy syndrome (OAS), showed similar cell counts during and outside the pollen season (not shown), and patients with asthma had elevated IgE positive cells (p = 0.017) (median: 151 cells/mm^2^) in the duodenal mucosa during the pollen season (not shown).

#### Patients without gastrointestinal symptoms (NS-group)

Also, in the NS-group, the duodenal biopsies showed significantly higher numbers of CD11c + dendritic cells (median: 3.55% of relative stained area) (p = 0.06) and a tendency for elevated eosinophil counts (p = 0.050) (median: 151.50 cells/mm^2^) and IgE-positive cells (p = 0.060) (median: 241 cells/mm^2^) during the pollen season as compared with the biopsy specimens taken off-season (Figure 4). In this group there was a correlation between total serum IgE and the number of IgE-positive cells in the biopsies taken outside the pollen season (r = 0.660, p = 0.020), but no significant correlation was found in the biopsies taken during the pollen season.

In the subgroup of patients with OAS there was an elevated frequency of duodenal tryptase positive cells (p = 0.037) (median: 120.50 cells/mm^2^) outside the pollen season (not shown). Only three patients in the NS group had asthma, which impaired further statistical analysis.

#### Intestinal allergic inflammation in asthmatics and non-asthmatics

When examining the whole material including patients from both S- and NS-group, no significant differences could be seen in the duodenal cell populations when comparing patients with or without asthma, regardless of season.

#### Healthy controls

In the healthy controls, there were no seasonal changes in any of the duodenal cell populations apart from the T cells that showed a slight but significant increase during the pollen season (p = 0.034) (Figure 4.)

### IgE reactivity against allergen components

To compare the total IgE reactivity between the S- and NS-group the sum of the ISU score for all PR-10 proteins from each patients was calculated in order to obtain a mean score for each group. The overall mean score of IgE reactivity to PR-10 proteins was significantly higher (p < 0.001) in the S-group 62.91 ISU (IQR 65.30; 95% CI 20.90-104.90) compared to NS-group 0.12 ISU (IQR 0.0; 95% CI -0.15-0.40). However, there were no significant differences in the IgE levels against the PR-10 proteins between the S and NS groups when analyzing the data separately for the different seasons. There was a trend towards an elevated median sensitization score in the S group to birch pollen related food 4.9 ISU (IQR 15.9; 95% CI 2.11-29.86) compared to NS group 2.75 ISU (IQR 9.65; 95% CI -0.5-18.5) in samples taken during the birch pollen season. The ISAC data with the sensitization patterns of the patients are shown in the Additional file 1: Table S1.

#### Patients with GI symptoms (S-group)

**Figure 5:**
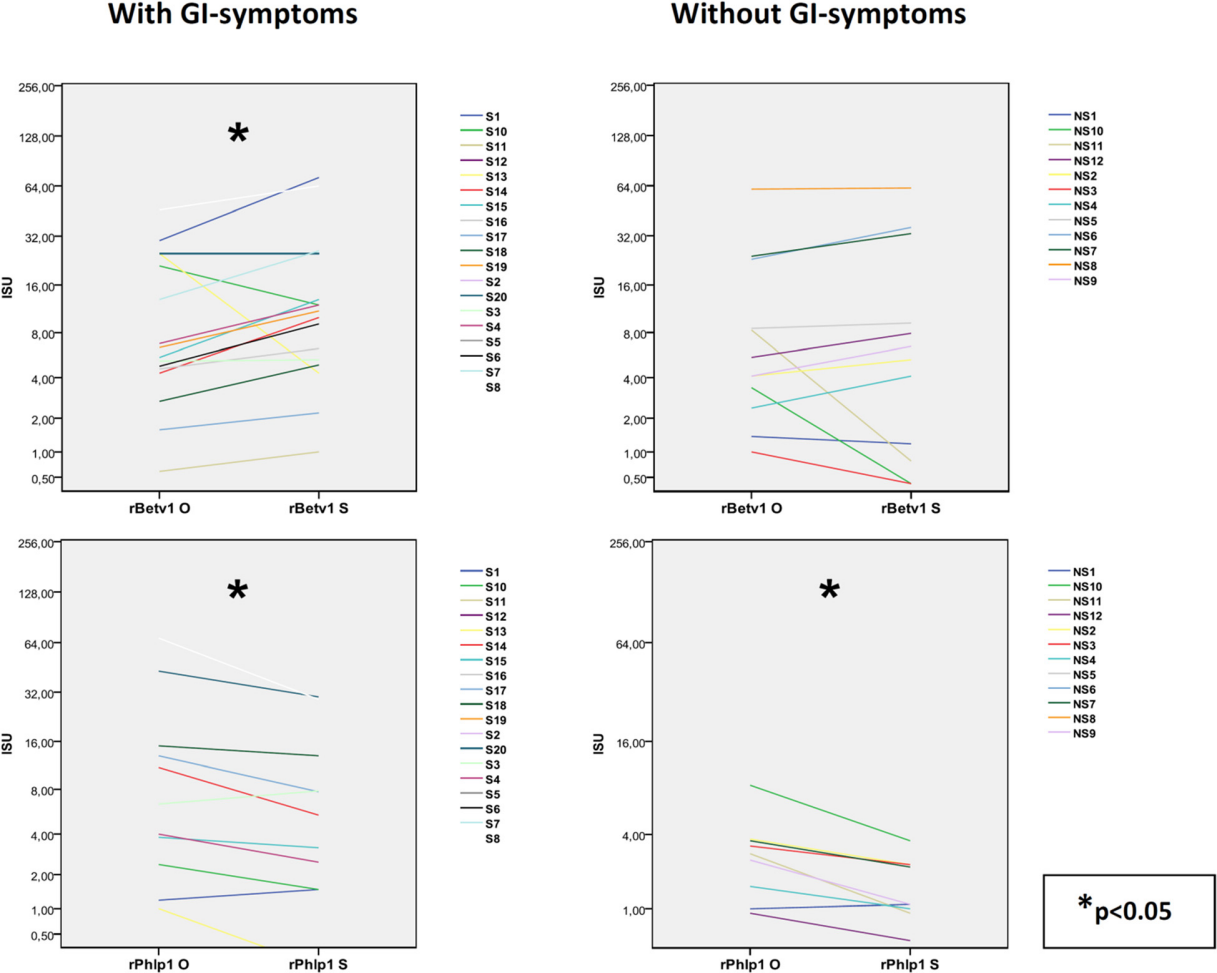
**Seasonal variation in IgE titers (ISU) to the major birch allergen rBetv 1 and the grass pollen antigen Phlp1.** Each colored line represents one patient and the left value is from outside the birch pollen season and the value to the right from samples taken during the birch pollen season (S= patients with GI symptoms, NS= patients without GI symptoms).

In this group we noted significantly higher levels of IgE to the major birch pollen allergen (rBet v 1) (p = 0.047), hazel pollen (rCor a 1.0101) (p = 0.014), and apple (rMal d 1) (p = 0.041) in blood samples taken during the birch pollen season. Outside the birch pollen season there was an elevated IgE reactivity to timothy pollen rPhl p 1 (p = 0.016) (Figure 5).

There were no significant differences in the IgE reactivity to any other ISAC component regardless of pollen season. In the subgroup of patients with OAS there was a significantly elevated IgE reactivity to the pollen antigens both during and outside the pollen season for rBet v 1 (p = 0.020, resp p = 0.006) and rAln g 1 (alder) (p = 0.010, resp p = 0.002), as well as to the related food antigens, rMal d 1 (p = 0.044, resp p = 0.029), rPru p 1 (peach) (p = 0.002, resp p = 0.002), rAra h 8 (peanut) (p = 0.014, resp p = 0.022), rCor a 1.04 (hazelnut) (p = 0.012, resp p = 0.004).

In this group the median average IgE reactivity to all PR-10 proteins was 21 ISU (IQR 33; 95% CI 9.42-64.08), and to birch- related foods 4.9 ISU (IQR 15.9; 95% CI 2.11-29.86) in samples taken during the pollen season, compared with 11 ISU (IQR 46.2; 95% CI 10.11-42.21) and 2.9 ISU (IQR 15; 95% CI 3.48-18.33) respectively, in samples taken off-season. The results concerning the mean score of ISAC components are provided in the Additional file 1: Table S1. The IgE reactivity to PR-10 proteins in the serum samples correlated significantly with the numbers of eosinophils in biopsies taken during the pollen season and with the total IgE levels in serum both during and outside the pollen season (r = 0.63 p = 0.004 and r = 0.67, p = 0.002 respectively). A positive correlation was also found between CD11c + cells in biopsies taken during the pollen season and IgE for rCor a 1.0101 (r = 0.61 p = 0.006) and rAra h 8 (r = 0.50 p = 0.028).

There was no correlation between IgE reactivity to components of the PR-10 proteins and the symptoms displayed by each patient neither during nor outside the pollen season.

#### Patients without GI symptoms (NS-group)

In asymptomatic patients there was no correlation between the degree of intestinal inflammation and the reactivity to PR-10 proteins. In this group of patients there was a significantly higher IgE reactivity to grass pollen rPhl p 1 (p = 0.010) and rMal d 1 (p = 0.018) in blood samples taken during the birch pollen season and in rAln g 1 (p = 0.002), nAct d 8 (p = 0.018) and rGly m 4 (p = 0.017)in samples taken outside the birch pollen season. In the subgroup of OAS patients we found a significantly elevated IgE reactivity to some pollen and pollen related components like rCor a 1.0101 (p = 0.022), rPru p 1, rAra h 8 (p = 0.036) in samples taken during the pollen season, and to rCor a 1.0101 (p = 0.013), to rPru p 1(p = 0.037) as well as r Ara h 8 (p = 0.020) in samples taken off-season. The median of average IgE reactivity to all PR-10 proteins was 18.9 ISU (IQR 65.18; 95% CI 3.6-89.7), and to birch-related foods 2.75 ISU (IQR 9.65; 95% CI -0.5-18.5) in samples taken during the birch pollen season, compared to 9.65 ISU (IQR 39.75; 95% CI 1.9-49.5) and 2.8 ISU (IQR 7.15; 95% CI -0.62-14.8), respectively, in samples taken outside the pollen season. The results showing the mean score of ISAC components are provided in the Additional file 1: Table S1.

#### Healthy controls

None of the healthy controls showed IgE reactivity to any of the allergens tested.

## Discussion

The objective of this study was to elucidate whether subjective gastrointestinal symptoms in birch pollen allergic patients are related to the degree of allergic inflammation in their intestinal mucosa, and to monitor if the intestinal allergic inflammation varies with the pollen season. Pollen allergic patients with gastrointestinal symptoms were compared with birch pollen allergic patients without any subjective and self-reported gastrointestinal disturbances and both these groups were compared with a group of healthy individuals. The results show that birch-pollen allergic patients with gastrointestinal symptoms, display a significant intestinal allergic inflammation with elevated numbers of eosinophils, IgE-positive cells, CD3+ T cells and CD11c + dendritic cells during the pollen season. This confirmed our previous findings [1]. The design of the present study with a larger patient sample than the previous study, allowed us to discriminate between patients with or without subjective GI symptoms and revealed that the intestinal allergic inflammation was obvious also in asymptomatic patients Also in these patients the pathology was aggravated during the birch pollen season. Furthermore, in both patients with and without GI symptoms, we observed elevated levels of IgE to most of the PR10-proteins during the pollen season. This was particularly pronounced in patients with OAS, indicating that cross reactions to birch related food may play a role in their pathology, which is supported by the “trigger foods” causing GI symptoms in OAS [21]. In addition, we observed that patients with allergic asthma were more prone to duodenal allergic inflammation as compared with non-asthmatic patients. The reason for this is unclear but could be due to a more severe allergy with greater engagement of the common mucosal immune system.

An elevated production of IgE antibodies against pollen allergens [22] during the birch pollen season is well established, but there are very few studies exploring the sensitization pattern to birch pollen related foods in relation to the birch pollen season [16]. Interestingly, high prevalence of clinical reactions to fruits and vegetables has been shown in birch pollen-sensitized patients with even higher prevalence in multi pollen-sensitized patients, which supports the notion that ingestion of pollen-related foods may act as an eliciting factor for allergic symptoms from different organs [23]. Reactivity to allergen components may identify allergens sharing similar structures, enabling detection of different levels of co-recognition by allergen-specific IgE antibodies e.g. Bet v 1 homologous allergens from hazelnut, apple and celery or vicilins from different legume seeds [16,17,24]. We found that IgE levels against the major birch allergen rBet v 1 as well as birch pollen related food items, are clearly increased during the birch pollen season in both groups of birch pollen allergic patients. In addition we observed that the IgE levels to some of the birch-pollen related foods like apple and peach, but also peanut, were significantly higher in patients with OAS. These findings support the hypothesis that ingestion of food items that are related to birch pollen might have a significant role in the allergic inflammation of the intestinal mucosa [19,25]. This may suggest that ingestion of birch pollen related food items during the birch pollen season could precipitate the onset of the gastrointestinal symptoms observed in pollen allergic patients. In support of this a recent study by Pickert et al. showed that 81% of patients with birch pollinosis and GI-symptoms, display a wheal and flare reaction in the gastrointestinal mucosa after colonoscopic allergen provocation with Bet v 1. Interestingly, a similar mucosal reaction was observed in 22% of birch pollen allergic patients with pollinosis who did not experience any GI-symptoms [26].

In the present study the patients in the S-group were found to have a continuous intestinal eosinophilia not related to the pollen season, which may indicate that they react to ingested pollen related food. This is supported by the positive correlation between eosinophil counts in in-season biopsies and the IgE reactivity to the PR-10 proteins during the pollen season. It is interesting though that birch-pollen allergic patients without gastrointestinal symptoms have a significantly increased intestinal eosinophilia during the pollen season. This may suggest a reaction to the pollen itself rather than pollen related food items, with a dissemination of the inflammation within the common mucosal immune system. A similar phenomenon has been observed in patients with eosinophilic esophagitis, where signs of a generalized subclinical eosinophilic inflammation at mucosal sites were part of the pathology [27,28]. Eosinophilic infiltration of the esophageal mucosa in patients with respiratory tract allergy has also been noted during the period of pollen allergy symptoms [28].

A number of clinical studies suggest a possible link between atopy and the augmentation of gastrointestinal symptoms during the birch pollen season in patients allergic to pollen [7,29,30], but few have addressed the cause of these GI symptoms [26]. Interestingly, in the present study the most frequent GI symptoms reported were abdominal distension, gases, pain, diarrhea, and constipation, which are common symptoms in patients diagnosed with IBS. Mast cells have been proposed to be included in the pathogenesis of IBS and increased numbers of mast cells have been found in intestinal mucosal biopsies in patients with IBS [31-34]. In line with these results we found a significantly higher number of IgE positive as well as tryptase-positive cells in the group of patients with GI symptoms compared to controls. In a more recent study examining both allergic and non-allergic patients with IBS, there was a higher number of eosinophils in the intestinal biopsies from patients with both atopy and IBS [5]. These results indicate that a number of patients with an allergic inflammation in the GI tract are diagnosed with IBS, which may hinder a correct diagnosis of their illness.

One possible limitation in the present study is that the patients were recruited during three consecutive years with variable severity of the seasonal pollen exposure, and that the individual natural exposure to pollen also varies according to living habits. However, the birch pollen seasons during the study years 2008–2010 were of representative severity for the study area without extreme variations (see supplement).

## Conclusions

In conclusion, we show here that regardless of subjective gastrointestinal symptoms, patients allergic to birch pollen have clear signs of an ongoing allergic inflammation in their intestinal mucosa, which is aggravated during the pollen season. Furthermore, patients who experience GI symptoms show somewhat elevated IgE levels to PR-10 proteins compared to the asymptomatic patients, which could be associated with the intake of birch pollen related food items.

## Abbreviations

S-group: Birch pollen allergic patients with gastrointestinal symptoms; NS-group: Birch pollen allergic patients without gastrointestinal symptoms; C-group: Control; CRD: Component-resolved diagnostic tests; GI: Gastrointestinal; OAS: Oral allergy syndrome; ISU: ISAC standardized units; SPT: Skin-prick test.

## Competing interests

The authors declare that they have no competing interests.

## Authors’ contributions

All authors were involved in the discussions and contributed to writing the document. All authors read and approved the final manuscript.

## Supplementary Material

Additional file 1 The questionnaire used for grading the symptoms in the group of patients with gastrointestinal symptoms. Click here for file
