# Supplementary material for: Intestinal allergic inflammation in birch pollen allergic patients in relation to pollen season, IgE sensitization profile and gastrointestinal symptoms
Source: Clin Transl Allergy. 2014 May 30;4:19. doi: 10.1186/2045-7022-4-19 (PMC4048541; doi:10.1186/2045-7022-4-19)
Supplement: Additional file 1 — The questionnaire used for grading the symptoms in the group of patients with gastrointestinal symptoms. [file 2045-7022-4-19-S1.doc]

**The questionnaire used for grading the symptoms in the group of patients with gastrointestinal symptoms**

1. Do you feel any pain or discomfort in your stomach?

- - - YES
    - NO

**2.** If, YES, do you usually experience this pain or discomfort:

- - - during the day? ________________________________________________________
    - more frequently during a certain period of the year? ___________________________
    - at meal-times?__________________________________________________________

***3.*** What kind of gastrointestinal symptoms do you experience?

- Burning sensation/reflux
- Nausea
- Vomiting
- Stomach ache
- Pain in the lower bowel
- Cramping or aching
- Bloating
- Flatulence
- Diarrhea
- Constipation
- Pain that starts always at meal-times
- Pain that does not appear in connection with meal-times
- Pain that disappears with defecation after a bowel motion
- Other (specify)? ______________________________________________

**4.** Do you find that your gastrointestinal symptoms increase during one or more of the following seasons?

- - - Spring
    - Summer
    - Autumn
    - Winter

**5.** Do you avoid any type of food during the season(s) when you experience symptoms?

- - - YES
    - NO

**6.** Do you experience increased breathing difficulties or asthma symptoms in connection with/at the same time as the increased gastrointestinal symptoms?

- - - YES
    - NO

**Supplementary figure**

Birch and grass pollen counts (pollen grains/m^3^) during 2007-2010 in the area where the patients were recruited.

**Supplementary table**

IgE sensitization profile as analyzed by ISAC for the two groups. (S=with symptoms, NS=without symptoms) the figure depicts the number of patients in each group with the indicated reactivity and the mean reactivity to the PR10-proteins during the pollen season in these patients. IgE reactivity analysed with ISAC is provided in ISU units.

| PR-10 | Major allergen | S ISAC  (n=20) mean score | NS  (n=12) | ISAC mean score |
| --- | --- | --- | --- | --- |
| rBet v 1 | Birch | PR-10 protein 17 13.9 | 12 | 14 |
| rAln g 1 | Alder | PR-10 protein 16 3.8 | 8 | 1.2 |
| rCor a 101.01 | Hazel | PR-10 protein 15 3 | 7 | 0.8 |
| rAct d 8 | Kiwi | PR-10 protein 0 0 | 0 | 0 |
| rApi g 1 | Celery | PR-10 protein 2 0 | 2 | 0.2 |
| rDau c 1 | Carrot | PR-10 protein 0 0 | 0 | 0 |
| rMal d 1 | Apple | PR-10 protein 11 3.5 | 7 | 2.6 |
| rPru p 1 | Peach | PR-10 protein 12 2.7 | 9 | 2 |
| rAra h 8 | Peanut | PR-10 protein 12 2.3 | 7 | 1 |
| rCor a 104.01 | Hazelnut | PR-10 protein 14 5.8 | 7 | 2.8 |
| rGly m 4 | Soybean | PR-10 protein 6 0.4 | 2 | 0.2 |
| Other | Allergen |  | S  (n=20) | NS (n=12) |
| nCyn d 1 | Bermuda Grass | Grass Group 1 | 9 | 9 |
| rPhl p 1 | Timothy Grass | Grass Group 1 | 11 | 9 |
| rPhl p 2 | Timothy Grass | Grass Group 2 | 7 | 3 |
| nPhl p 4 | Timothy Grass | Berberine Bridge enzyme | 8 | 9 |
| rPhl p 5 | Timothy Grass | Grass Group 5 | 9 | 6 |
| rPhl p 6 | Timothy Grass | Grass Group 6 | 6 | 3 |
| rPhl p 7 | Timothy Grass | Calcium binding 2EF-hand pr. | 0 | 0 |
| rPhl p 11 | Timothy Grass | Ole e-1-related protein | 5 | 1 |
| rPhl p 12 | Timothy Grass | Profilin | 1 | 0 |
| rBet v 2 | Birch | Profilin | 2 | 1 |
| rBet v 4 | Birch | Calcium binding 2EF-hand pr. | 0 | 0 |
| nCry j 1 | Japanese cedar | Pectate lyase | 0 | 0 |
| nCup a 1 | Cypress | Pectate lyase | 0 | 0 |
| nOle e 1 | Olive | Common Olive group 5 | 1 | 0 |
| nOle e 2 | Olive | Profilin | 1 | 1 |
| rPla a 1 | Plane Tree | Putative invertase inhibitor | 0 | 0 |
| nPla a 2 | Plane Tree | Polygalacturonase | 0 | 0 |
| nAmb a 1 | Ragweed | Pectate lyase | 0 | 0 |
| nArt v 1 | Mugwort | Defensin | 3 | 1 |
| nArt v 3 | Mugwort | nsLTP | 0 | 0 |
| rPar j 2 | Wall pelitory | nsLTP | 0 | 0 |
| nSal k 1 | Saltwort | Pectin methylesterase | 0 | 0 |
| rMer a 1 | Annual Mercury | Profilin | 2 | 1 |
| nAct d 1 | Kiwi | Cysteine protase | 1 | 0 |
| nAct d 2 | Kiwi | Thaumatin-like protein | 0 | 0 |
| nAct d 5 | Kiwi | Kiwellin | 0 | 0 |
| nPru p 3 | Peach | nsLTP | 0 | 0 |
| rAna o 2 | Cashewnut | Legumin-like protein | 0 | 0 |
| nAra h 1 | Peanut | Storage protein 7S globulin | 0 | 0 |
| nAra h 2 | Peanut | Storage protein conglutin | 0 | 0 |
| nAra h 3 | Peanut | Storage protein 11S globulin | 0 | 0 |
| rBer e 1 | Brazil nut | Storage protein 11Sglobulin | 0 | 0 |
| rCor a 8 | Hazelnut | nsLTP | 0 | 0 |
| rCor a 9 | Hazelnut | Storage protein 11Sglobulin | 0 | 0 |
| rGly m 5 | Soybean | Storage protein Beta-conglycin | 0 | 0 |
| rGly m 6 | Soybean | Storage protein Glycin | 0 | 0 |
| nSes i 1 | Sesame seed | Storage protein 2S albumin | 0 | 0 |
| nTri a 18 | Wheat | Agglutinin isolectin 1 | 0 | 0 |
| nTri a Gliadin | Wheat | Crude gliadin | 0 | 0 |
| nTri a190101 | Wheat | Omega-5-gliadin | 0 | 0 |
| nTria a A_TI | Wheat | Alpha-Amylase/Trypsin inhibitor | 0 | 0 |
| rHev b 1 | Latex | Rubber elongation factor | 0 | 0 |
| rHev b 3 | Latex | Smal rubber particle protein | 0 | 0 |
| rHev b 5 | Latex | Acidic protein | 0 | 0 |
| rHev b 6 | Latex | Hevein | 0 | 0 |
| rHev b 8 | Latex | Profilin | 2 | 1 |
| nAna c 2 | Bromelin | CCD marker | 0 | 0 |
| nBos d 4 | Milk | Alpha-lactalumin | 0 | 0 |
| nBos d 5 | Milk | Beta-lactoglobulin | 0 | 0 |
| nBos d 6 | Milk | Serum albumin | 0 | 0 |
| nBos d 8 | Milk | Casein | 0 | 0 |
| nBosd_lactoferin | Milk | Transferin | 0 | 0 |
| nGal d 1 | Egg | Ovomucoid | 0 | 0 |
| nGal d 2 | Egg | Ovalbumin | 0 | 0 |
| nGal d 3 | Egg | Conalbumin | 0 | 0 |
| nGal d 5 | Egg | Serum albumin | 0 | 0 |
| rCyp c 1 | Carp | Parvalbumin | 0 | 0 |
| rGad c 1 | Cod | Parvalbumin | 0 | 0 |
| rPen a 1 | Shrimp | Tropomyosin | 1 | 0 |
| rPen i 1 | Shrimp | Tropomyosin | 1 | 0 |
| nPen m 1 | Shrimp | Tropomyosin | 1 | 0 |
| nDer f 1 | D. farinae | Cysteine protease | 1 | 3 |
| rDerf2 | D. farinae | NPC2 family | 2 | 3 |
| nDer p 1 | D. pteronissinus | Cysteine protease | 2 | 3 |
| nDer p 2 | D. pteronissinus | NPC2 family | 2 | 3 |
| rDer p 10 | D. pteronissinus | Tropomyosin | 1 | 0 |
| rEeur m 2 | Storage mite | NPC2 family | 1 | 2 |
| rCan f 1 | Dog | Lipocalin | 1 | 1 |
| rCan f 2 | Dog | Lipocalin | 0 | 0 |
| rCan f 3 | Dog | Serum albumin | 0 | 0 |
| nEqu c 3 | Horse | Serum albumin | 0 | 0 |
| rFel d 1 | Cat | Uteroglobin | 6 | 4 |
| nFel d 2 | Cat | Serum albumin | 0 | 0 |
| rFel d 4 | Cat | Lipocain | 0 | 1 |
| nMus m 1 | Mouse | Lipocain | 0 | 0 |
| rAlt a 1 | Altenaria | Acidic glycoprotein | 1 | 0 |
| rAlt a 6 | Altenaria | Enolase | 0 | 0 |
| rAsp f 1 | Aspergillus | Mitogillin family | 0 | 0 |
| rAsp f 2 | Aspergillus | Fibrinogen binding protein | 0 | 0 |
| rAsp f 3 | Aspergillus | Peroxysomal protein | 3 | 0 |
| rAsp f 4 | Aspergillus | Unknown | 0 | 0 |
| rAsp f 6 | Aspergillus | Mn superoxide dismutase | 2 | 0 |
| rCla h 8 | Cladosporium | Mannitol dehydrogenase | 0 | 0 |
| nApi m 1 | Honey bee | Phospholipase A2 | 1 | 0 |
| nApi m 4 | Honey bee | Melittin | 0 | 0 |
| rBla g 1 | Cockroah | Cockroah group 1 | 0 | 0 |
| rBla g 2 | Cockroah | Aspartic protease | 0 | 0 |
| rBla g 4 | Cockroah | Calycin | 0 | 0 |
| rBla g 5 | Cockroah | Glutathione S-transferase | 0 | 0 |
| rBla g 7 | Cockroah | Tropomyosin | 1 | 0 |
| rAni s 1 | Anisakis | Serine protease inhibitor | 0 | 0 |
| rAni s 3 | Anisakis | Tropomyosin | 1 | 0 |
